# Design and Experimental Research of a Miniature Digital Hydraulic Valve

**DOI:** 10.3390/mi9060283

**Published:** 2018-06-04

**Authors:** Junhui Zhang, Meisheng Yang, Bing Xu

**Affiliations:** State Key Laboratory of Fluid Power and Mechatronic Systems, Zhejiang University, Hangzhou 310058, China; benzjh@zju.edu.cn (J.Z.); yms1986@126.com (M.Y.)

**Keywords:** micro digital valve, mechanical structure, magnetic circuit, switching characteristics, digital hydraulic system

## Abstract

A digital hydraulic valve is an important component of the digital hydraulic system, and its performance is directly related to the system function. In order to make the valve system more competitive in dimension, digital valve miniaturization is an important research point. A new micro digital valve is designed, which is analyzed from the mechanical structure and magnetic circuit mechanism, and the design difficulties are also expounded. The four subsystems and switching characteristics of the valve are theoretically analyzed and simulated. In order to test the performance of the valve, a test system is designed, and performance of the new micro valve is tested. The test results show that the switch characteristic analysis of the valve is correct. The comparison between the test curve and the simulation curve is carried out, which demonstrates that the accuracy of the simulation model is reasonable. The theoretical analysis of the new micro digital valve is consistent with experiments.

## 1. Introduction

Compared with the traditional proportional/servo technology, the digital hydraulic system has advantages of anti-pollution, high redundancy, fast response and so on [1,2,3]. The key component of the digital hydraulic technology is the digital valve. In order to make the digital valve have greater competitive advantage in flow resolution and response speed, the research of digital valve focuses on miniaturization. Among them, the micro bistable valve, memory alloy valve and mini electromagnetic valve have been studied deeply.

A small bistable valve with outer diameter of 31 mm was developed [4]. This miniature valve uses permanent magnet materials. During conversion state of the valve, electricity makes the upper half valve part (or the lower half valve part) form the magnetic field to obtain the electromagnetic force, so that the valve is opened or closed. After the state transition, it is not necessary to maintain the state of the valve, which saves energy in the process of valve operation. The opening energy is only 11 mJ, and the opening time is 1.6 ms and the closing time is 3.2 ms under the condition of 21 MPa supply pressure and 10 MPa pressure differential.

**Figure 1:**
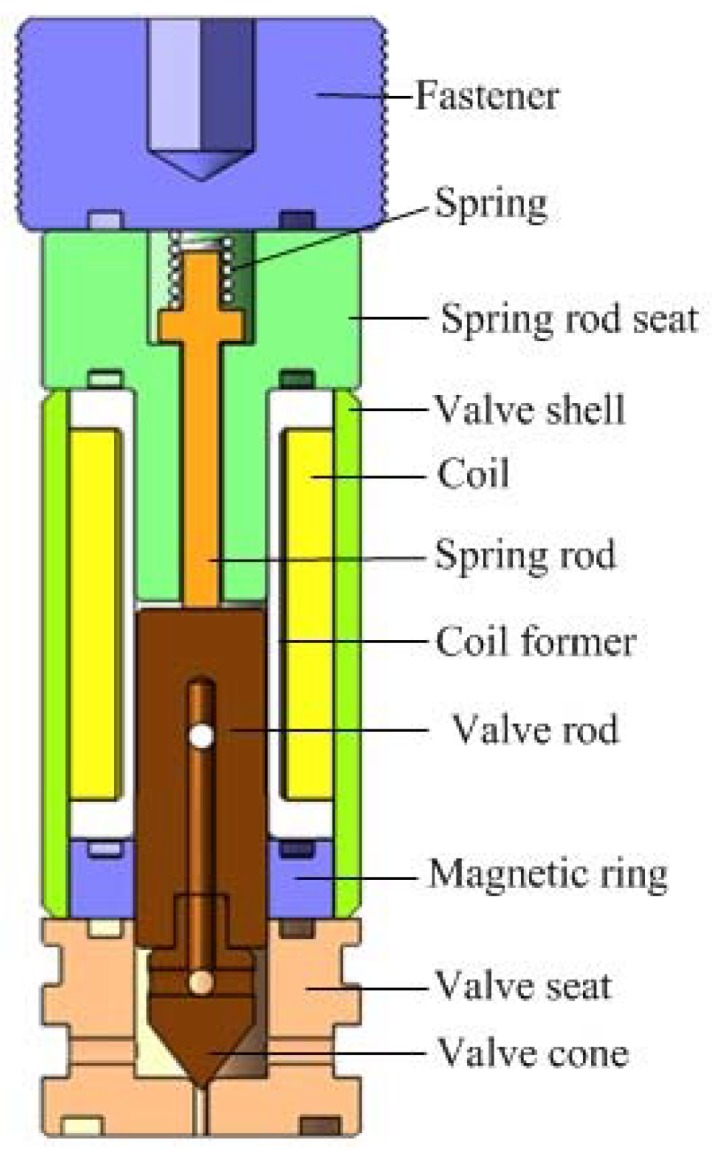
Structure diagram of the micro digital valve.

The micro bistable valve was further miniaturized. The outer diameter of the new valve was 18.5 mm [5,6]. The three-dimensional structure diagram of the bistable valve and its physical map can be seen in Ref. [5] (Figures 1 and 7). The response time of the valve is less than 2 ms, and the maximum continuous operation frequency is greater than 50 Hz. Under the pressure differential of 1 MPa, the nominal flow rate is 3.3 L/min, and the maximum pressure differential is 21 MPa. Although the energy consumption of the bistable valve is small, the overall size of the micro valve is larger than that of the solenoid valve because of the complex inner structure. 

The structure of miniature memory alloy valve is proposed by the Oak Ridge National Key Laboratory (Oak Ridge, TN, USA) [7]. The miniature memory alloy valve structure can be seen in Ref. [7] (Figure 3) and physical map can be seen in Ref. [8] (Figure 6). Outer diameter of the miniature memory alloy valve is 3 mm. The miniature valve spool used a memory alloy material (SMA), which enables the memory alloy material to produce small deformation and expansion in the direction of length by input voltage, realizing quick opening and closing of the valve. The miniature memory alloy valve is applied to the control of prosthetic finger joints [8]. The dynamic response of the valve can reach 200 Hz. The flow rate is 10 mL/min, which is suitable for special medical devices.

**Figure 2:**
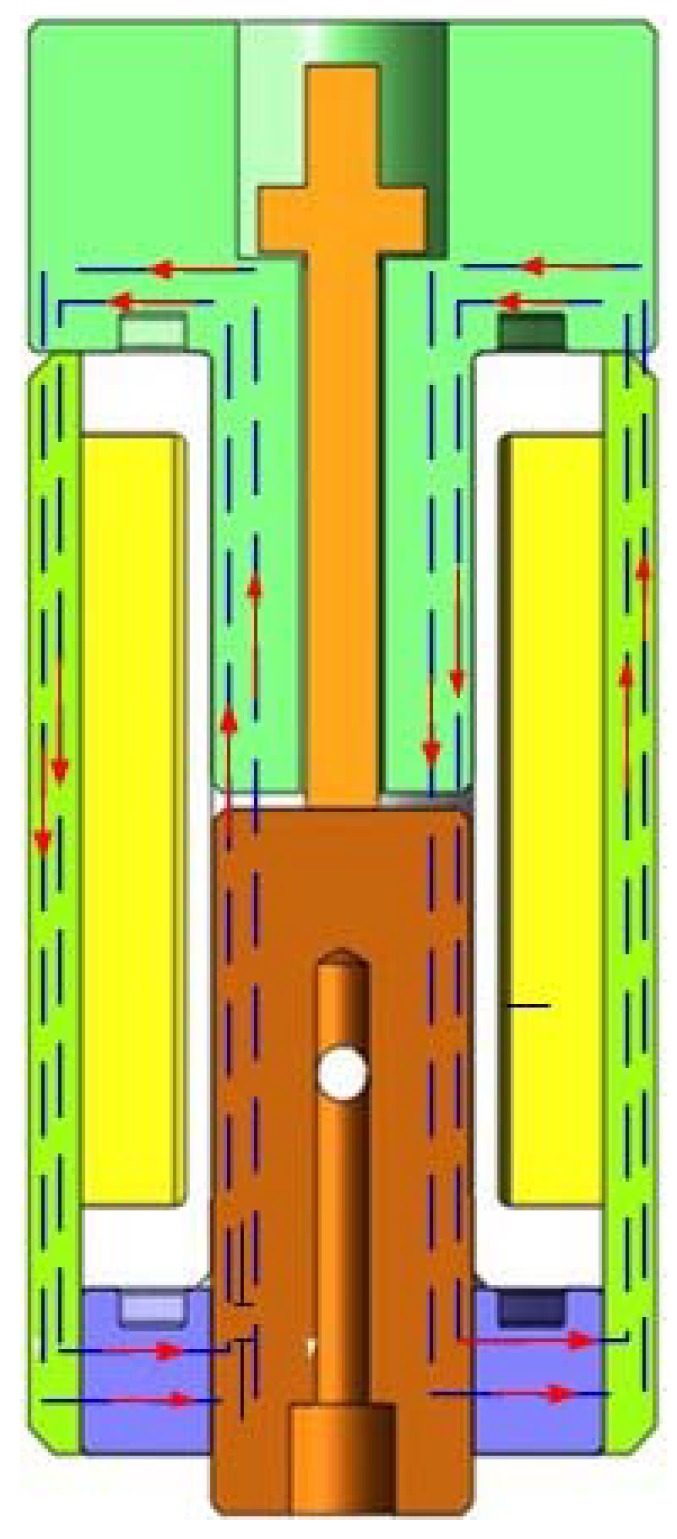
Magnetic circuit diagram of the digital valve.

Some scholars have studied the amplification mechanism of micro piezoelectric valve [9]. The physical map and structure of the miniature piezoelectric valve can be seen in Ref. [9] (Figures 1 and 2). The driving voltage is applied to the piezoelectric crystal material, causing small displacement deformation. The deformation is amplified by lever action. By this means, the valve is opened or closed. Because the assembly error and thermal deformation of the piezoelectric valve had a great influence on the overall performance of the valve, the compensation mechanism of the amplification mechanism was studied, and the sensitivity of the structural parameters was deeply analyzed.

**Figure 3:**
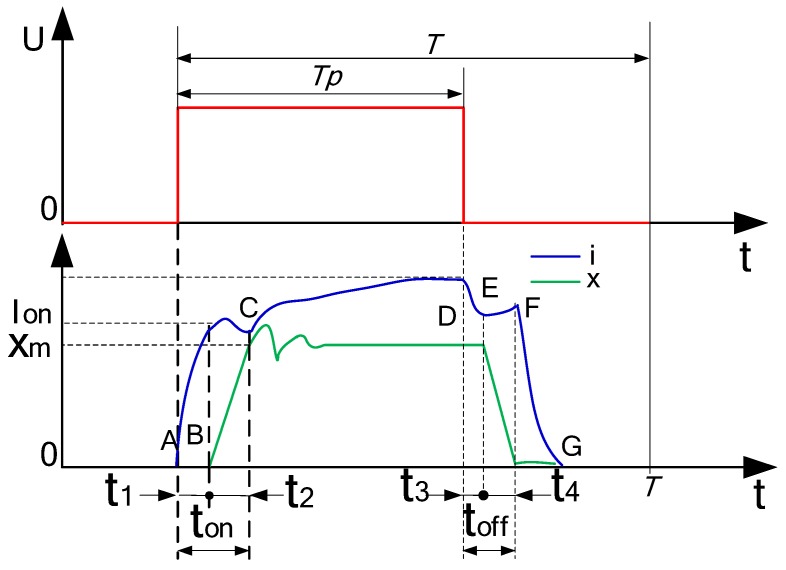
Signal–displacement relation diagram of digital valve.

Some scholars have studied the miniaturization of digital valves, and developed the first generation of miniature electromagnetic valve, whose outer diameter is 10 mm [10]. The first generation of micro solenoid valve can be seen in Ref. [10] (Figure 8). In the study, electromagnetic field and excitation circuit were optimized, and the reasonable matching parameters were obtained [11]. Due to cavitation generated under large pressure differential, the pressure setting is less than 12.5 MPa, the opening time is 1.9 ms, and the closing time is 2.2 ms. In the magnetic circuit, because of the use of stainless steel materials with poor magnetic isolation performance, there is a “circuit breaker”. In order to avoid reducing the permeability, the electrical pure iron was treated as the end of the valve core, whose hardness was low, so the durability of the valve core was poor [12]. Compared with the hardened treatment of the valve core end, the reasonable area of boronizing hardening treatment was optimized. The second generation of micro electromagnetic valve with outer diameter of 11 mm has been developed in 2011 [13], which can be seen in Ref. [13] (Figure 17). Compared with the first generation of solenoid valve, the electromagnetic field of the mini electromagnet has been further optimized, and the opening energy consumption of the valve has been reduced to 57 mJ. The closing response time is 2.8 ms, and the opening response time is 2.2 ms. Pressure differential has great influence on closing delay time of the valve. The third generation miniature electromagnetic valve with outside diameter of 10 mm has been developed in 2012 [14]. The third generation of miniature solenoid valve can be seen in Ref. [14] (Figure 5). The valve has been greatly improved in the assembly process, and the coil is supported by a stainless steel skeleton. Under 21 MPa test pressure, the opening response time is 2 ms, and the closing time response is 2.8 ms. The fourth generation of solenoid valve with outer diameter of 10 mm has been developed in 2014 [15]. The fourth generation of micro solenoid valves can be seen in Ref. [15] (Figure 3). Compared with the stainless steel coil skeleton used in the third generation of micro valve, the fourth generation of a valve coil uses polyacetal resin material to prevent the magnetic circuit from breaking. Cobalt iron alloys are used in soft magnetic materials. The opening delay time is 1.4–2.3 ms under different pressure drop, and the closing delay time is 2 to 3.4 ms.

A miniature solenoid valve suitable for oil and gas wells under the high temperature and high-pressure mining environment was developed by Zhejiang University [16]. The miniature solenoid valve can be seen in Ref. [16] (Figure 16). Considering the special working conditions, the size of the micro valve is 20 mm, the maximum working pressure is 21 MPa and the flow rate is 2 L/min by the optimization of the valve body material and the matching of the parameters of the electromagnetic field. The return spring of the valve was designed in the middle of the miniature solenoid valve. The returned spring of the novel micro digital hydraulic valve discussed in this paper is designed at the top of the valve. By this means, the percentage of magnetic flux area can be increased. 

Table 1 presents characteristics of these micro valves. The limitations of the valves are listed in Table 1. In order to overcome the disadvantages, we can improve the characteristics by design of the rational magnetic materials and magnetic circuit in Section 2.

## 2. Design of the Valve

Analogous to the micro valve size of the Tampere University of Technology, the outer diameter of valve presented in this paper is less than 15 mm, and the length size is less than 40 mm. Pressure flow characteristics: 0.7 L/min@3.5 MPa; dynamic characteristic: switching response time is less than 1.5 ms.

The micro digital valve is used in the digital hydraulic system. In order to achieve accurate flow control and less energy loss, the cone valve type is more reasonable. In order to achieve a quick opening, the stiffness coefficient of the reset spring should not be too large, so as to ensure the acceleration of spool motion during opening. It is also a difficult point for the electromagnetic design, which should not be too small; otherwise, the flow is too small and the pressure loss is too large. The main dimensions and parameters of the micro valve are listed in Table 2.

An important index of a digital valve is dynamic response speed, which depends on the magnitude of the electromagnetic driving force. A reasonable electromagnetic structure can make small volume electromagnets have larger driving potential. The returned spring of the novel micro digital hydraulic valve is designed at the top of the valve to increase the magnetic flux intercepting area in the magnetic circuit, and the magnetic flux intercepting area is positively related to the size of the electromagnetic force. In this way, the greater electromagnetic force will be generated under the same input energy to increase the response speed. The miniature digital valve structure diagram is shown in Figure 1. Table 3 is a list of materials for each component of the miniature solenoid valve. Figure 2 shows the magnetic circuit diagram of the digital valve. In the structure of the valve, a magnetic ring of the soft magnetic material is designed, which replaces the non-magnetic material, shortens the length of the magnetic circuit and reduces the overall reluctance loss.

The valve spool adopts segmented welding structure, including two sections: spool rod and valve core cone. The valve spool rod uses the DT4 soft magnetic material to make the magnetic line pass through the valve spool, and the valve core cone uses Cr12MoV, which is one wear-resistant and has high hardness material. Thus, the valve spool can resist the deformation during the continuous impact process, and ensure the sealing performance of the cone valve.

## 3. The Mathematical Model of the Valve

The micro digital valve contains four subsystems coupled with each other. The solenoid subsystem focuses on the calculation of current intensity of coil winding, magnetic flux density and electromagnetic force. The solenoid subsystem imports voltage and exports electromagnetic force. The mechanical subsystem integrates electromagnetic force and spring force, and outputs the displacement and velocity. The fluid subsystem describes the force on the spool, flow rate and outlet pressure. The chamber pressure dynamic subsystem depicts the relation of the hydraulic compressibility and the pressure change [19]. The main characteristic equations of each subsystem of the solenoid valve can be expressed as follows:

### 3.1. Electromagnetic Subsystem

The electromagnetic subsystem consists of an electrical and magnetic circuit. The balance equation for the subsystem can be written by the Kirchhoff’s law:

(1) $$U=Ri(t)+\frac{d(N\Phi (t))}{dt},$$

where *U* represents input voltage, *R* represents electrical resistance, *N* represents coil turns number, Φ represents flux and *i* represents current of the circuit. The current of the coil generates magnetic flux, and then produces force and displacement. It can be expressed as:

(2) $$Ni=\frac{2B_{g}x_{t}}{\mu }+\sum H_{i}l_{i},$$

where $\frac{2B_{g}x_{t}}{\mu }$ represents the magneto-motive force necessary to establish the flux in the air gap, and $\sum H_{i}l_{i}$ represents the magneto-motive force necessary to establish the flux in the iron parts of the circuit. $B_{g}=f(x,i)$ is the air gap flux density, and $x_{t}$ is the total air gap distance.

The relationship between electromagnetic force and magnetic flux can be expressed as:

(3) $$F_{e}=\frac{\Phi^{2}}{A\mu }=\frac{B_{g}^{2}A}{\mu },$$

where *A* is effective cross-sectional area of the magnetic core, the magnetic flux Φ is a function of current *i* and movement distance of the valve spool. Therefore, the electromagnetic force equation can be written as:

(4) $$F_{e}=\frac{\mu A{\left(Ni\right)}^{2}}{4{\left(x_{t}-x\right)}^{2}}.$$

The dynamic motion of armature can be derived as:

(5) $$m_{a}{\ddot{x}}_{a}=F_{e}-b_{a}{\dot{x}}_{a}-K_{s}(x_{s}+x_{a}),$$

where $m_{a}$ is armature mass, $x_{a}$ is armature distance, $b_{a}$ is viscous friction coefficient, $K_{s}$ is spring stiffness and $x_{s}$ is pre-compression length of solenoid spring.

### 3.2. Mechanical Subsystem

The micro digital valve adopts the poppet valve structure, and the spool is subjected to the hydraulic pressure force, electromagnetic force, spring force, viscous friction force and flow force, which can be expressed as:

(6) $$F_{h}+F_{e}-F_{v}-F_{f}-K_{s}(x_{s}+x_{v})=m_{v}{\ddot{x}}_{v},$$

(7) $$F_{v}=\frac{\pi \cdot d_{v}\cdot L_{v}\cdot \mu_{o}}{c_{r}}\cdot x_{v},$$

where $F_{h}$ is hydraulic pressure force, $F_{v}$ is viscous friction force, $F_{f}$ is flow force, $\mu_{o}$ is dynamic viscosity of oil, $m_{v}$ is spool mass, $x_{v}$ is spool distance, $c_{r}$ is radial clearance and $d_{v}$ is spool diameter.

### 3.3. Fluid Subsystem

In the flow rate calculation, the flow condition is evaluated by the Reynolds number. The Reynolds number can be calculated using the following equation:

(8) Re=qhdHAvvh.

The valve orifice area and hydraulic diameter of the valve orifice can be expressed as:

(9) $$A_{v}=\pi \cdot D\cdot x_{v}\cdot \sin\alpha -\pi \cdot x_{v}^{2}\cdot \sin^{2}\alpha \cdot \cos\alpha ,$$

where *α* is half cone angle:

(10) $$d_{H}=2x_{v}\cdot \sin\alpha .$$

The flow rate from the control valve orifice can be expressed as:

(11) q={cq⋅Av⋅sign(Δp)⋅Δpρh   for Re≥Rec 2Cql⋅Av⋅dHvh⋅ρh⋅(Δp)    for Re<Rec,

where *ρ_h_* is density of the hydraulic oil, ∆*p* is pressure differential, Re_c_ is critical Reynolds number and *C_ql_* is valve discharge coefficient for laminar flow.

The steady flow force can be calculated by momentum change, as presented in Equation (12):

(12) $$F_{f}=C_{q}\cdot \pi \cdot d_{s}\cdot x_{v}\cdot \Delta p\cdot \sin(2\alpha ).$$

### 3.4. Chamber Dynamic Pressure Subsystem

The chamber pressure can be calculated by the basic hydraulic compressibility equation:

(13) $${\dot{p}}_{c}=\frac{B}{V_{c}}(q_{c}\pm A_{c}{\dot{x}}_{c}).$$

The positive or negative sign in this equation depends on the direction of the valve motion.

### 3.5. Research on Switching Characteristics

Switching characteristics refer to the relationship between spool displacement and time under the action of the pulse-width modulation (PWM) signal. Static characteristics refer to the relationship between the average displacement of valve core ${\bar{x}}_{v}$ and the duty cycle of PWM signal τ. Definition:

$${\bar{x}}_{v}=\int_{0}^{T}x_{v}dt/Tx_{m},$$

where *x_m_* represents maximum displacement of the spool.

Therefore, the actual average flow rate of the digital valve is as follows:

(14) $$\bar{Q^{\prime }}=DC_{d}\omega \bar{x_{v}}\sqrt{\frac{2}{\rho }(P_{p}-P_{T})}.$$

The actual average valve core opening degree is: $\bar{x_{v}}=\int_{0}^{T}x_{v}dt/Tx_{m}$.

The mathematical description of the displacement static characteristics of the valve core is:

(15) $${\bar{x}}_{v}=\{\begin{array}{ll} 0 & \tau \in [\begin{array}{cc} 0, & \tau_{1}) \end{array}\\ \frac{1}{2\tau_{2}}(1+\frac{\tau_{4}}{\tau_{2}}){\left(\tau -\tau_{1}\right)}^{2}+\frac{\tau_{3}}{\tau_{2}}\left(\tau -\tau_{1}\right) & \tau \in [\begin{array}{cc} \tau_{1}, & \tau_{on}) \end{array}\\ \tau +\frac{1}{2}(\tau_{4}-\tau_{2})+(\tau_{3}-\tau_{1}) & \tau \in [\begin{array}{cc} \tau_{on}, & 1-\tau_{off}) \end{array}\\ 1-\frac{1}{2\tau_{4}}(1+\frac{\tau_{2}}{\tau_{4}})(1-\tau -\tau_{3})-\frac{\tau_{1}}{\tau_{4}}(1-\tau -\tau_{3}) & \tau \in [\begin{array}{cc} 1-\tau_{off}, & 1-\tau_{3}) \end{array}\\ 1 & \tau \in [\begin{array}{cc} 1-\tau_{3}, & 1] \end{array} \end{array},$$

where $t_{on}=t_{1}+t_{2}$; $\tau_{on}=t_{on}/T$; $t_{off}=t_{3}+t_{4}$; $\tau_{off}=t_{off}/T$; 

When $T_{p}\leq t_{1}+t_{2}$, the valve spool is sucking, but the clutch release is not in the end, and the release delay of the spool will be less than $t_{3}$.

**Figure 4:**
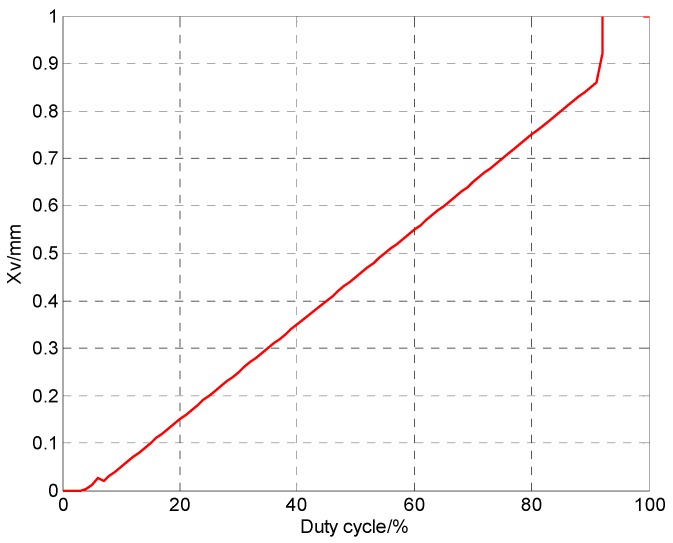
Signal–displacement simulation chart of digital valve.

When $T_{p}\geq T-t_{3}-t_{4}$, the spool is too late to release, the delay of spool core will be less than $t_{1}$, where, $t_{1}$ is spool opening delay time; $t_{2}$ is spool opening time; $t_{3}$ is valve core closing delay time; $t_{4}$ is valve core closing time; $t_{on}$ is the sum of the opening delay time and opening time of the valve core; $t_{off}$ is the sum of the closing time and closing delay time of the valve core; The relationship between the signal and displacement of a digital valve is shown in Figure 3. Point A refers to the start point of the current rising (voltage rising), and point B refers to the start point of the displacement increase. Time between point A and point B refers to opening response delay of the micro valve. Point C refers to the point where the displacement of the valve is the maximum displacement. At the moment, the inflection point of current appears. Point D refers to the start point of the current falling (voltage falling). Point E refers to the start point of the displacement decrease. Time between point D and point E refers to closing response delay of the micro valve. Point F refers to the point of zero displacement. Point G refers to the point of zero current of the micro valve. MATLAB 2010 simulation software (MathWorks, Natick, MA, USA) is used for calculation, and the result is shown in Figure 4. In the calculation, the range of 0–0.035 is taken as the first stage, when $\tau \in [\begin{array}{cc} 0, & \tau_{1}) \end{array}$; the range of 0.035–0.07 is as the second stage, when $\tau \in [\begin{array}{cc} \tau_{1}, & \tau_{on}) \end{array}$; the range of 0.07–0.92 is as the third stage, when $\tau \in [\begin{array}{cc} \tau_{on}, & 1-\tau_{off}) \end{array}$; the range of 0.92–0.985 is as the fourth stage, when $\tau \in [\begin{array}{cc} 1-\tau_{off}, & 1-\tau_{3}) \end{array}$ and the range of 0.985–1 is the fifth stage when $\tau \in [\begin{array}{cc} 1-\tau_{3}, & 1] \end{array}$.

## 4. Experimental Study on the Micro Digital Valve

### 4.1. Test System

**Figure 5:**
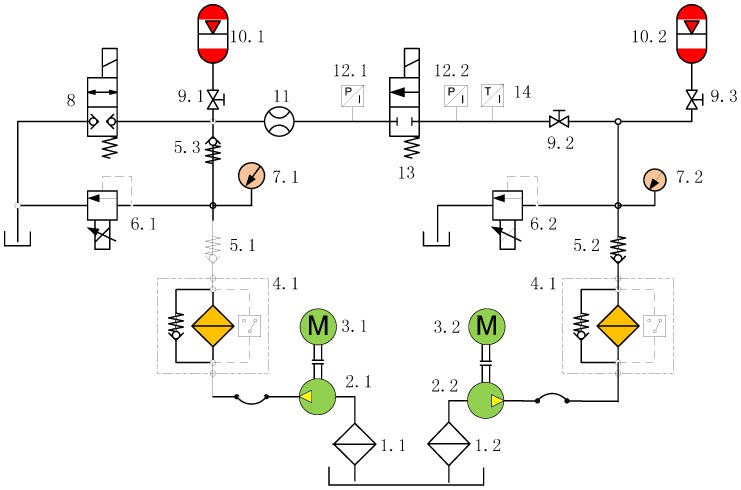
Schematic diagram of a micro digital valve test system. 1.1 and 1.2: Crude filter; 2.1 and 2.2: Hydraulic pump; 3.1 and 3.2: Motor; 4.1 and 4.2: Fine filter; 5.1, 5.2 and 5.3: Check valve; 6.1 and 6.2: Proportional relief valve; 7.1 and 7.2: Pressure meter; 8: Electromagnetic reversing valve; 9.1, 9.2 and 9.3: Cut-off valve; 10.1 and 10.2: Accumulator; 11: Flow meter; 12.1 and 12.2: Pressure sensor; 13: Miniature digital valve; 14: Temperature sensor.

**Figure 6:**
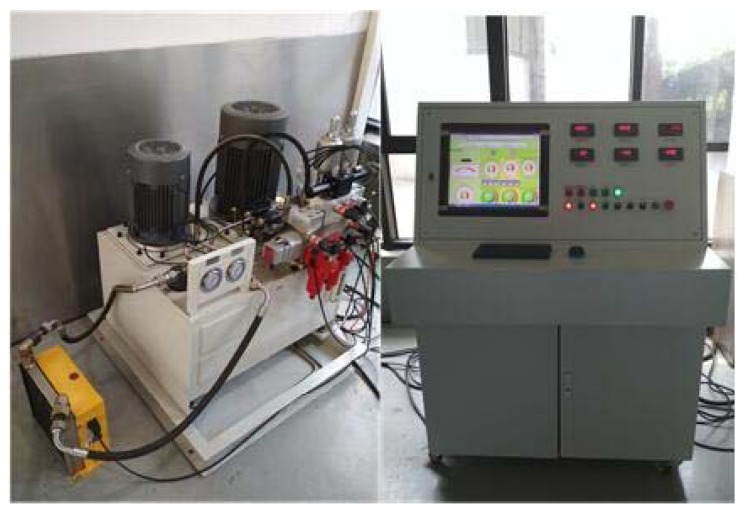
Micro digital valve test system.

**Figure 7:**
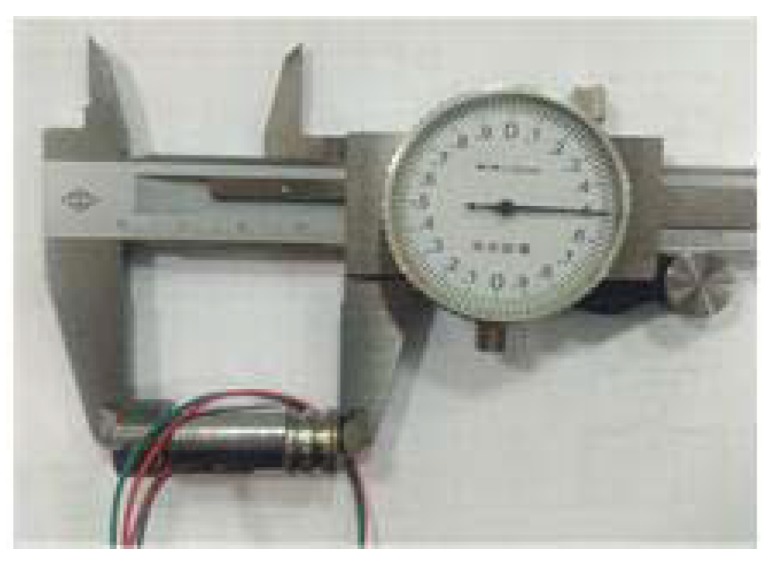
Miniature digital valve.

The principle of the micro digital valve test system is shown in Figure 5. The physical map of the test system and the micro digital valve are shown in Figure 6 and Figure 7, respectively. The system uses No. 46 hydraulic oil, and the working voltage is 12 V. The pressure of the front and the end of the valve is controlled by the proportional relief valve 6.1 and 6.2. The flow rate is detected by flowmeter 11, and the oil temperature is monitored by temperature sensor 14. The accumulators 10.1 and 10.2 reduce the pressure fluctuation of the system.

### 4.2. Static Characteristic Test of the Miniature Digital Valve

**Figure 8:**
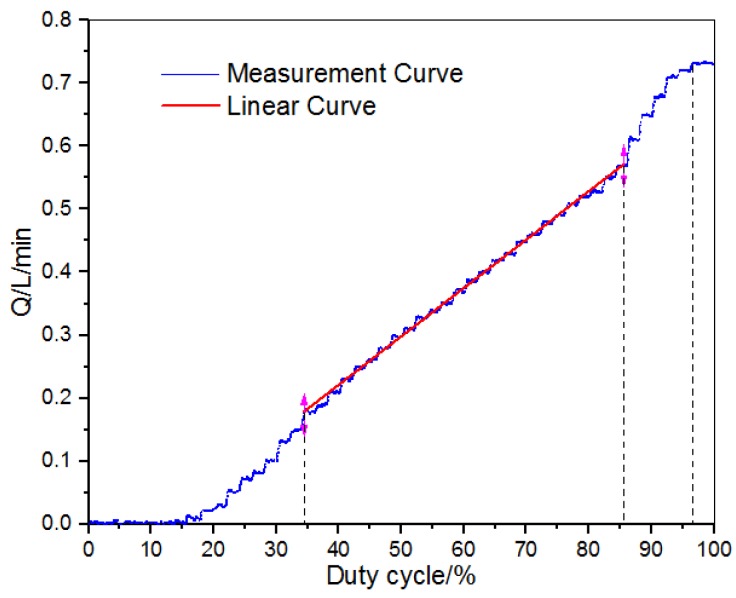
Signal–flow characteristic curve of miniature digital valve.

Figure 8 shows the signal–flow characteristic curve of the micro digital valve. Under the test condition of 50 bar pressure differential, the signal increased from 0 to 100%. When the signal is in the range of 0–15%, the miniature valve is in the dead zone position, so the valve spool does not move, the valve closes, and there is no flow output, which is equivalent to the first phase of the signal–displacement relationship. In the range of 15–34%, flow is in a nonlinear region, which is equivalent to the second stage of signal–displacement relation. The linearity area is within the range of 34–86%, and the linearity is good, which corresponds to the third stage of signal displacement relation. In the range of 86–97%, flow is in a nonlinear region, which corresponds to the fourth stage of signal displacement relation. In the range of 97–100%, flow reaches saturation state, which is equivalent to the fifth stage of signal displacement relation. Therefore, the relationship between signal–flow curve and signal–displacement curve corresponds. The correctness of the switch characteristics of the valve is verified.

**Figure 9:**
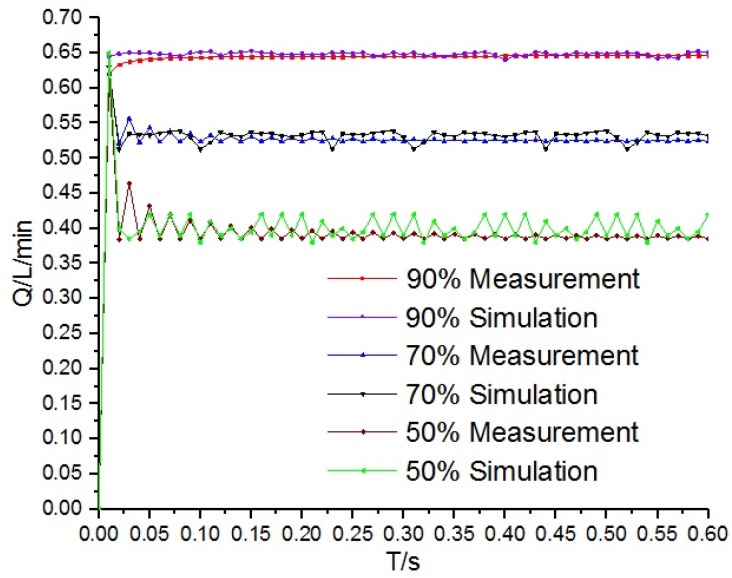
Simulation and test contrast curves.

Under the differential pressure of 35 bar and 100 Hz operation frequency, simulation and test of signal–flow is shown in Figure 9. Under the control signal of 50%, 70% and 90% duty ratio, the simulation curves of flow are compared with the test curve, which shows that the errors are within a reasonable range, and the simulation model is basically accurate.

**Figure 10:**
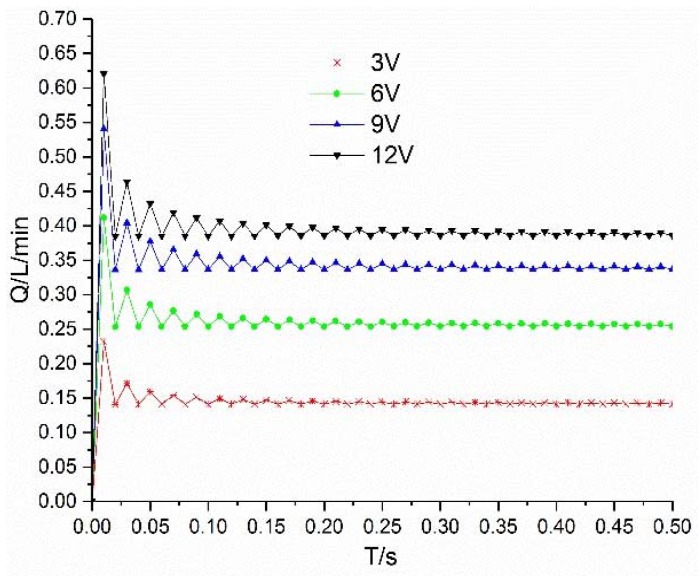
Flow curves under different voltages.

**Figure 11:**
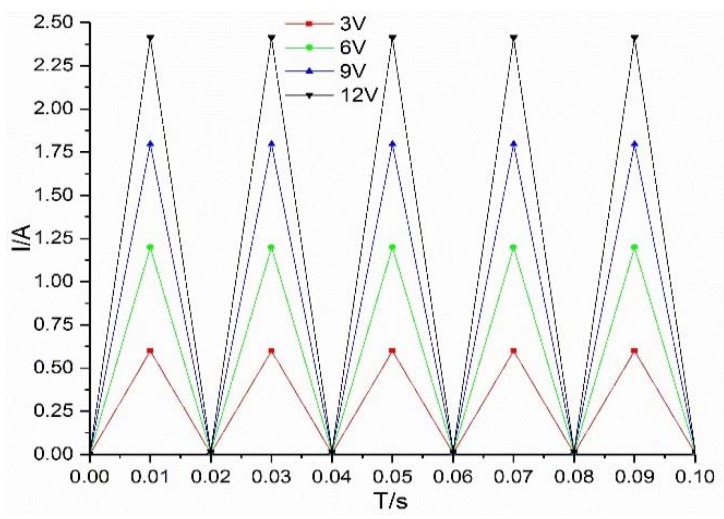
Current curves under different voltages.

The simulation curves of flow under different voltages are shown in Figure 10. Under the excitation of 3 V, 6 V, 9 V and 12 V, the flow rate increases with increase of voltage, but with the increase of voltage, the flow fluctuation also increases. This is because, with the increase of voltage, the fluctuation of valve working current increases, which eventually leads to the increase of flow fluctuation, as shown in Figure 11.

## 5. Conclusions

A new micro digital hydraulic valve is designed. Combined with design parameters, the structure and design difficulties of the valve are described. In order to realize the high speed of the micro digital valve, the magnetic circuit structure of the valve is mainly introduced:

(1) The mathematical modeling of the four subsystems of the valve is developed, by which the switching characteristics of the valve are analyzed theoretically. The signal–displacement relation is divided into five stages.
(2) A test system for testing the micro digital valve is designed, and the new micro valve test is carried out. The signal–flow curve is similar to the signal–displacement curve. It can also be divided into five stages to verify the correctness of switch characteristics.
(3) The accuracy of the mathematical model is verified by comparing the signal flow characteristic curves with the simulation results. The flow rate increases with the increase of duty cycle and input voltage.

## Figures and Tables

**Table 1:** Characteristics of the valves.

**Valve Type**	**Direct Operated Bistable Seat**	**Pilot Operated Seat**	**Direct Operated Bistable Spool**	**-**
**Valve Manufacturer**	**Tampere University of Technology**	**Tokyo Institute of Technology** [17]	**Sturman Industries** [18]	**-**
Nominal Flow @3.5 MPa	10 L/min	6 L/min	32 L/min	-
Max. Pressure Differential	21 MPa	14 MPa	n.a.	-
Size	31 mm × 28.2 mm	50 mm × 150 mm	110 mm × 35 mm × 35 mm	-
Disadvantage	Complex Structure	Lower Pressure	Larger Size	-
**Valve Type**	**Memory Alloy**	**Piezoelectric**	**Direct Operated Seat**	**Direct Operated Seat**
**Valve Manufacturer**	**Oak Ridge National Key Laboratory**	**Technische Universität Dresden**	**Tampere University of Technology**	**Zhejiang University**
Nominal Flow @3.5 MPa	10 mL/min	n.a.	1.4 L/min	2 L/min
Max. Pressure Differential	0.5 MPa	n.a.	21 MPa	21 MPa
Size	30 mm × 3 mm	n.a.	35 mm × 10 mm	45 mm × 20 mm
Disadvantage	Lower Pressure and Smaller Nominal Flow	Higher Voltage	Lower Reliability	Larger Size

**Table 2:** Dimensions and parameters of the micro valve.

Description	Dimensions and Parameters	Description	Dimensions and Parameters
Outer Diameter of the Valve	12 mm	Number of Coil Turns	300
Length of the Valve	36 mm	Diameter of Coil	0.18 mm
Outer Diameter of the Coil	10 mm	Air Gap Distance	0.3 mm
Length of the Coil	14 mm	Stiffness of the Spring	12 N/mm
Resistance	4.5 Ω	Diameter of Orifice	0.5 mm
Voltage	12 V	-	-

**Table 3:** Materials for each component of the miniature digital valve.

Part Name	Materials	Part Name	Materials	Part Name	Materials
Fastener	316L	Coil	Copper	Magnetic Ring	DT4
Spring	Stainless Steel	Spring Rod	316L	Valve Seat	Cr12MoV
Spring Rod Seat	DT4	Coil Former	316L	Valve Cone	Cr12MoV
Valve Shell	DT4	Valve Spool	DT4	-	-
